# Supplementary material for: Evaluation of chromatin accessibility in prefrontal cortex of individuals with schizophrenia
Source: Nat Commun. 2018 Aug 7;9:3121. doi: 10.1038/s41467-018-05379-y (PMC6081462; doi:10.1038/s41467-018-05379-y)
Supplement: Supplementary file 1 — Supplementary Information [file 41467_2018_5379_MOESM1_ESM.pdf]

Evaluation of chromatin accessibility in prefrontal cortex of individuals with schizophrenia

Supplementary Figures and Tables

Bryois et al.

Table of Contents

Supplementary Fig. 1: Peak merging statistics 2
Supplementary Fig. 2: ATAC-seq enrichment 3
Supplementary Fig. 3: ATAC-seq Fraction of Reads in Peaks in different tissues 4
Supplementary Fig. 4: Heritability enrichment for schizophrenia 5
Supplementary Fig. 5: Heritability enrichment from DNase-seq and ATAC-seq across 142 cell and tissue types 6
Supplementary Fig. 6: Differentially accessible peaks between cases and controls 7
Supplementary Fig. 7: Chromosomal interactions at the AS3MT locus where a cQTL co-localize with SCZ GWAS variants and eQTLs of both AS3MT and WBP1L). 8
Supplementary Fig. 8: Identification of open chromatin in heterogeneous tissue 9
Supplementary Fig. 9: Spearman rank correlations of biological replicates 10
Supplementary Fig. 10: Correlation between ATAC-seq principal components and exonic rate in RNA-seq. 11
Supplementary Fig. 11: Correlation matrix for imputed numerical metadata in 288 samples. 12
Supplementary Fig. 12: Association of PCs from imputed numerical metadata with diagnosis 13
Supplementary Fig. 13: Association of metadata with case-control status 14
Supplementary Fig. 14: Intercorrelations of numerical metadata significantly associated with case-control status 15
Supplementary Fig. 15: Postmortem interval and case-control status 16
Supplementary Fig. 16: Association of number of ATAC-seq peaks (1% FDR) with metadata 17
Supplementary Fig. 17: Correlation of technical covariates with number of peak calls 18
Supplementary Fig. 18: Coverage of ATAC-seq and DNase-seq datasets representing 125 cell types and tissues 19
Supplementary Fig. 19: Mean Jaccard index for ATAC-seq and DNase-seq data in 125 cell types and tissues 20
Supplementary Fig. 20: Jaccard index for ATAC-seq and DNase-seq data from 125 cell types and tissues 21
Supplementary Table 1: *Overlap of ATAC-seq data with external samples.* 22
Supplementary Table 2: *Enrichment of transcription factor binding motifs in DLPFC ATAC-seq peaks that overlap evolutionarily conserved sequences.* 23

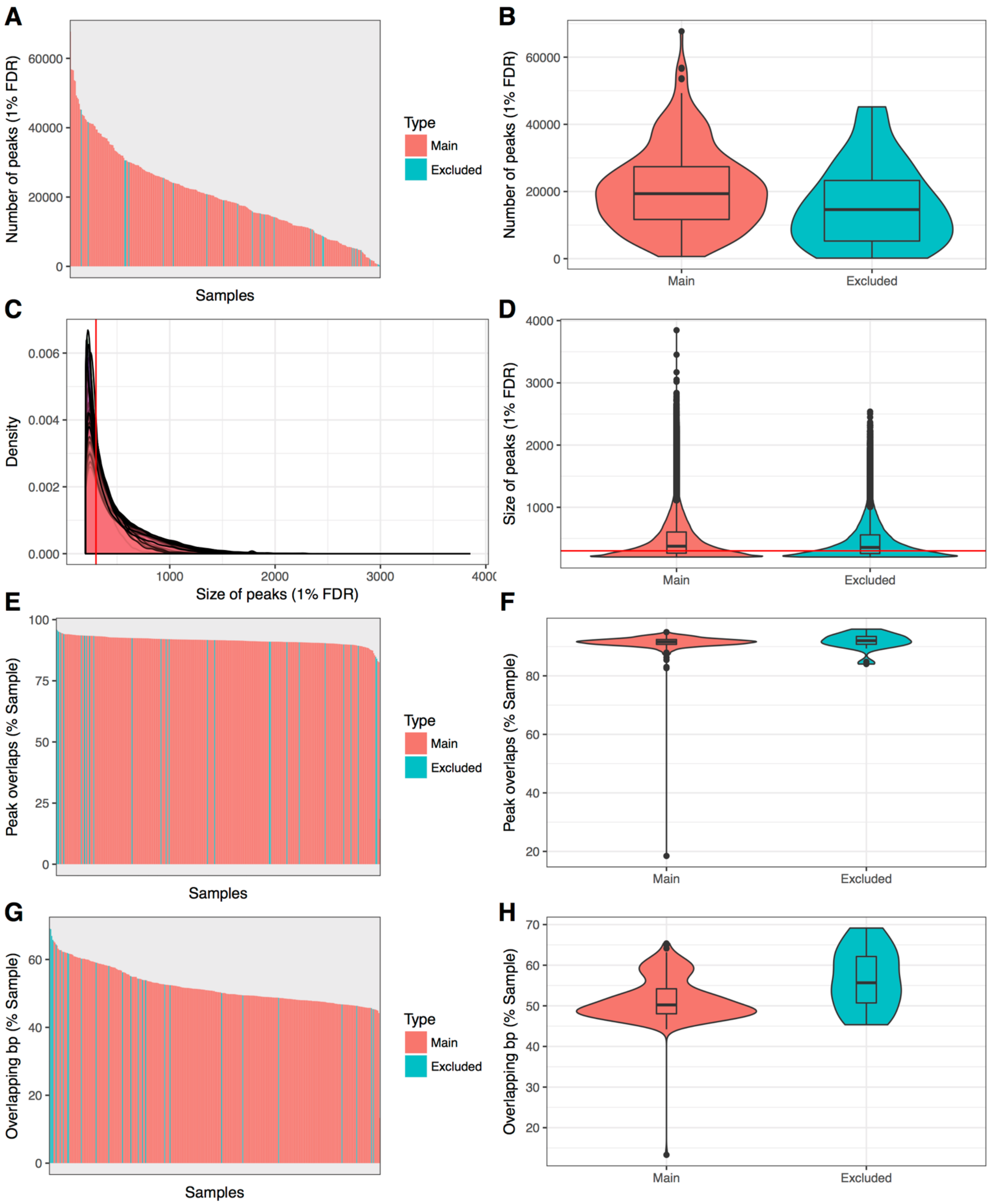

Supplementary Fig. 1: Peak merging statistics

(A) Number of peaks (1% FDR) for the 314 samples that were sequenced (Red: sample kept for peak merging (288), Blue: samples excluded from the peak merging (replicate or non-matching genotype) (B) and distribution. (C) Size of peaks (1% FDR) for the 314 sequenced samples, the red bar represents 300bp (D) and distribution. (E) Number of peaks of each sample overlapping the merged peak set (F) and distribution. (G) Percentage of base pairs of peaks samples that overlap the merged peak set (H) and distribution. The boxplots represents the following statistics: median (bolded line), the 1st and 3rd quartiles (bounds of box) and 1.5x the inter-quartile range (whiskers).

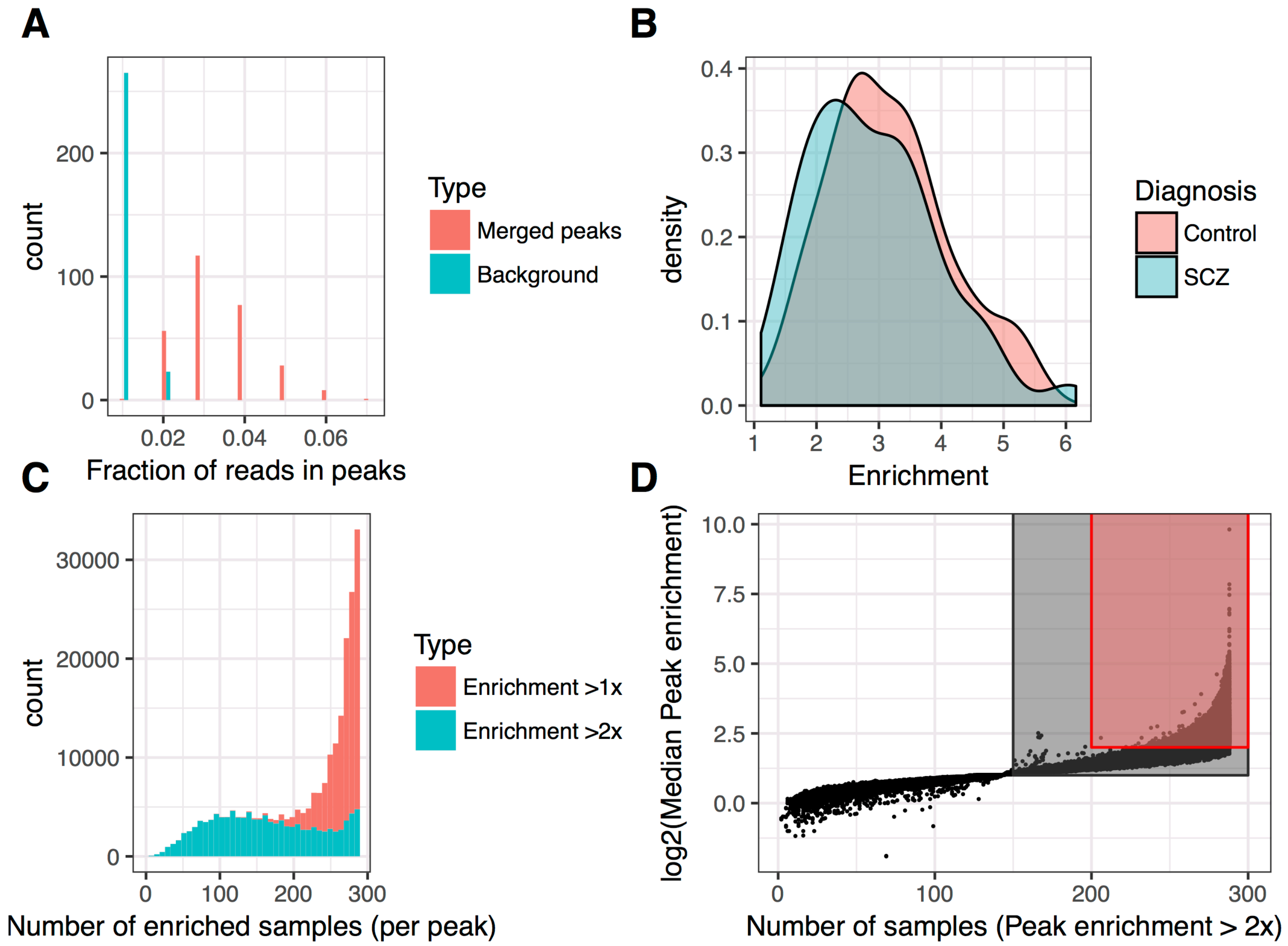

Supplementary Fig. 2: ATAC-seq enrichment

(A) Distribution of the fraction of reads mapping to merged peaks for each sample (red) compared to merged peaks randomly shuffled in the mapable genome (excluding ENCODE blacklisted regions and regions with a peak detected at 10% FDR in any of the 288 samples). (B) Density of ATAC-seq signal enrichment in the merged peaks (sum of reads in merged peaks/sum of reads in shuffled peaks per sample). (C) Distribution of the number of samples for which a peak is enriched at least 1x (red) or 2x (blue). (D) Median enrichment of peaks across samples in function of the number of samples for which the peak is enriched at least 2x. The grey and red rectangle highlight two sets of highly enriched peaks that were used to filter differential accessibility results to reduce the burden of multiple testing. These filters did not significantly increase in the number of peaks differently accessible due to age, post-mortem interval or diagnosis.

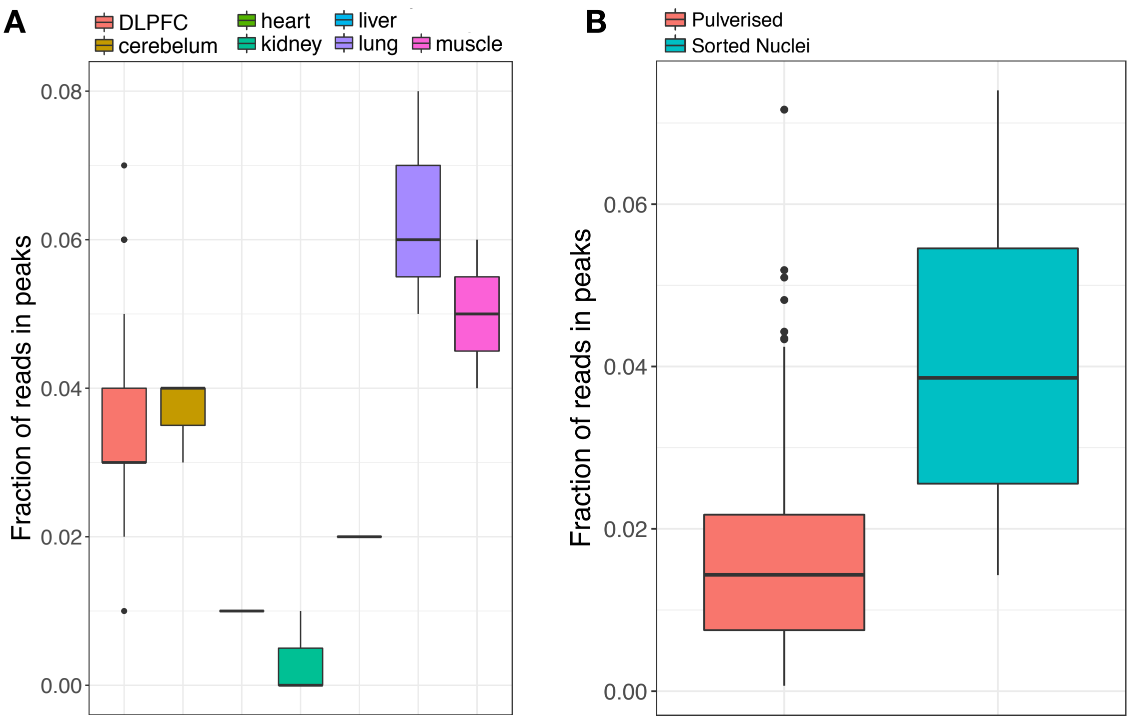

Supplementary Fig. 3: ATAC-seq Fraction of Reads in Peaks in different tissues

(A) Fraction of ATAC-seq reads mapping to merged peaks in the dorso-lateral prefrontal cortex (red) and other tissues. There are 288 samples for the DLPFC ATAC-seq and 3 samples for the 6 other tissues. (B) Fraction of ATAC-seq reads mapping to peaks detected at 1% FDR in each sample. The pulverized label represent our 288 samples while the sorted nuclei data were obtained from Fullard et al. [1] The boxplots represents the following statistics: median (bolded line), the 1st and 3rd quartiles (bounds of box) and 1.5x the inter-quartile range (whiskers).

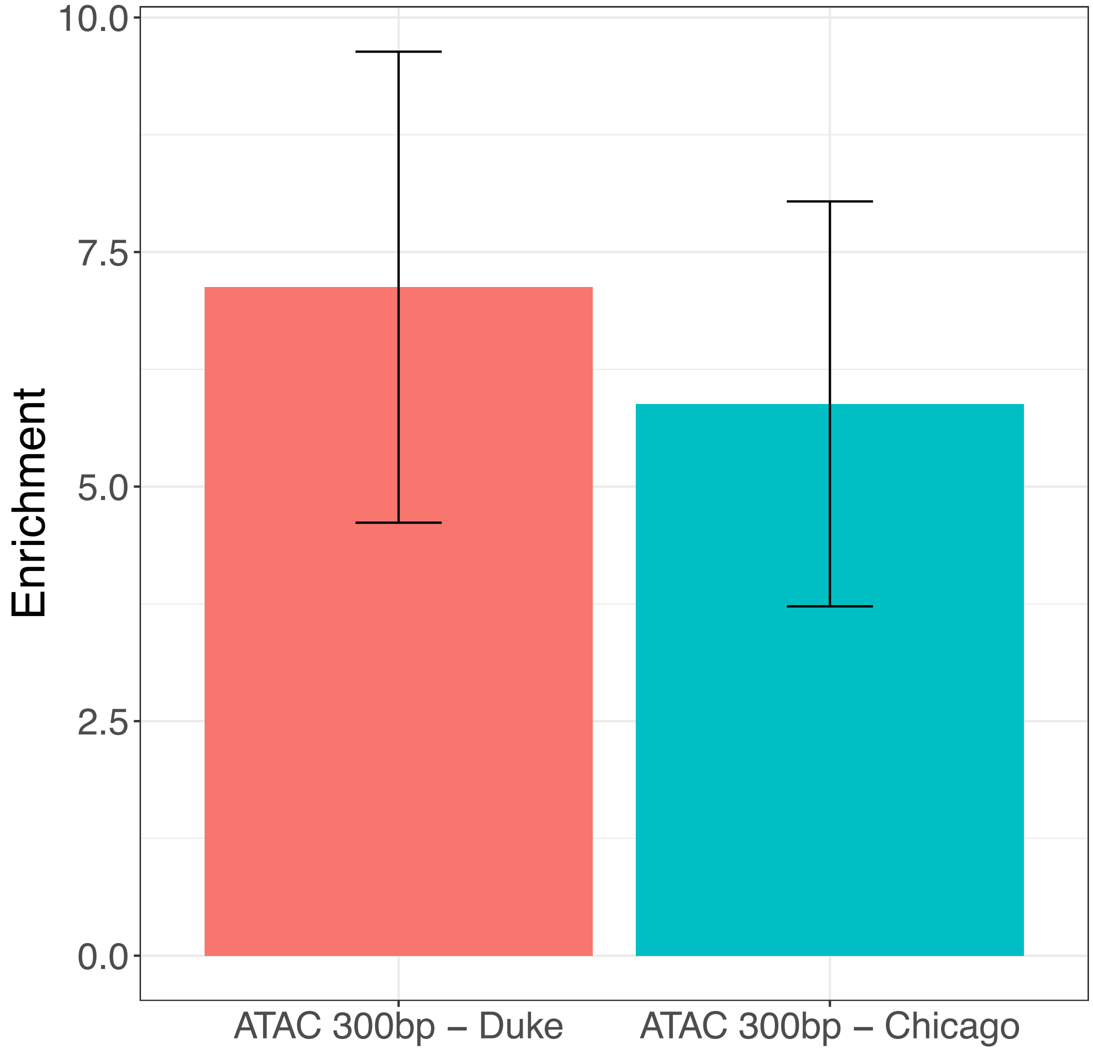

Supplementary Fig. 4: Heritability enrichment for schizophrenia

For the 118,152 ATAC-seq peaks analyzed in this study (Duke University) and replication in an independent ATAC-seq data set (University of Chicago) consisting of 157,660 peaks obtained using the same bioinformatics pipeline. The heritability enrichments were obtained using partitioned LD score regression using the full baseline model, the ATAC-seq peaks and an additional track with the ATAC-seq peaks extended on both sides by 500bp. Only the heritability enrichment (standard error) of the ATAC-seq peaks is shown here.

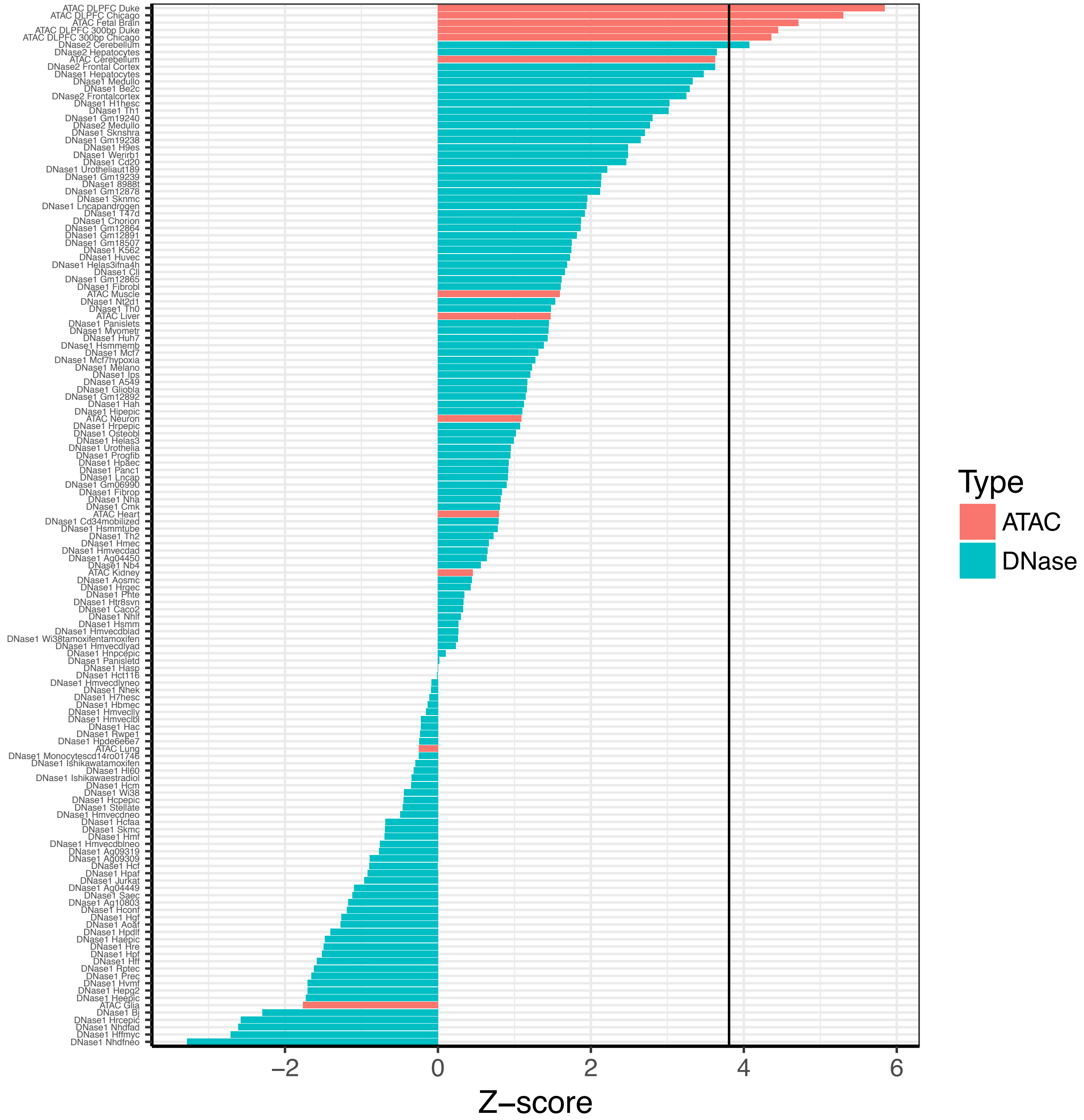

Supplementary Fig. 5: Heritability enrichment from DNase-seq and ATAC-seq across 142 cell and tissue types

Heritability enrichment Z-scores were determined from ATAC-seq (red) DNase-seq (blue) data generated from 142 cell types and tissues, including ATAC-seq data generated by Duke and Chicago (University of Illinois at Chicago, and the University of Chicago) using partitioned LD score regression. Each dataset was added to the full baseline model and tested one at a time. The black bar represents the Bonferroni significance threshold (p adjusted =0.01). Note that the top enrichment values were from ATAC-seq samples from Duke and University of Chicago.

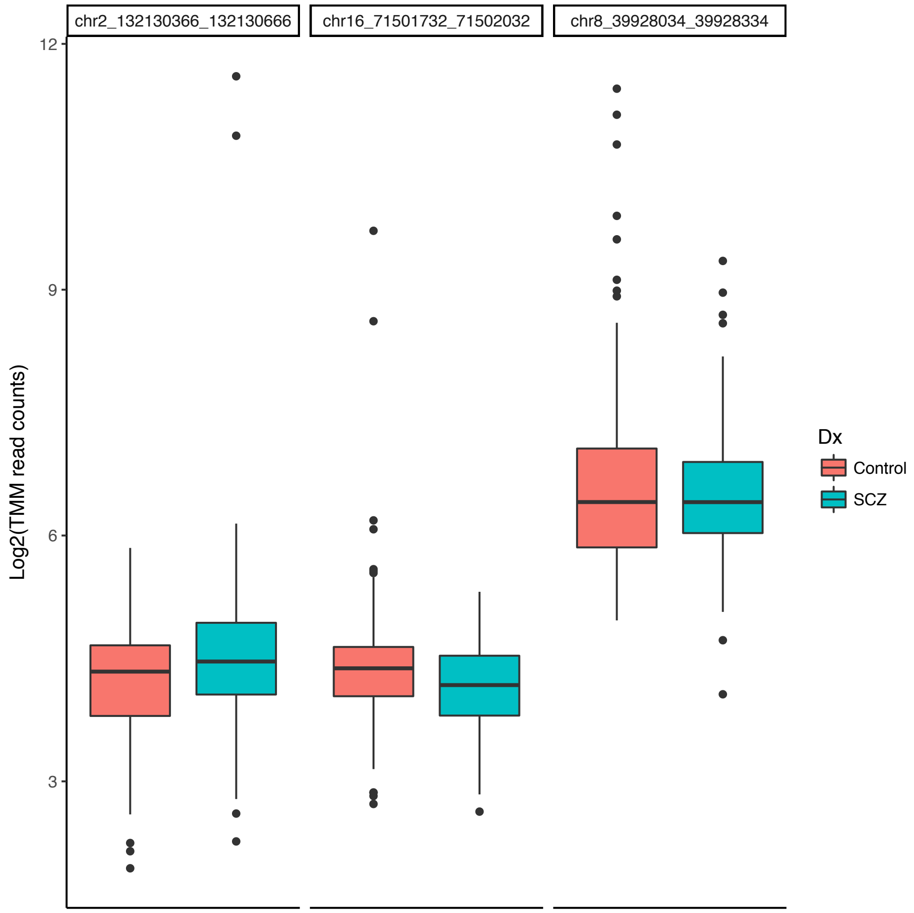

Supplementary Fig. 6: Differentially accessible peaks between cases and controls

Number of reads (log2) in cases and controls after TMM normalization[2] for the three peaks found to be significantly differentially accessible (5% FDR). The boxplots represents the following statistics: median (bolded line), the 1st and 3rd quartiles (bounds of box) and 1.5x the inter-quartile range (whiskers).

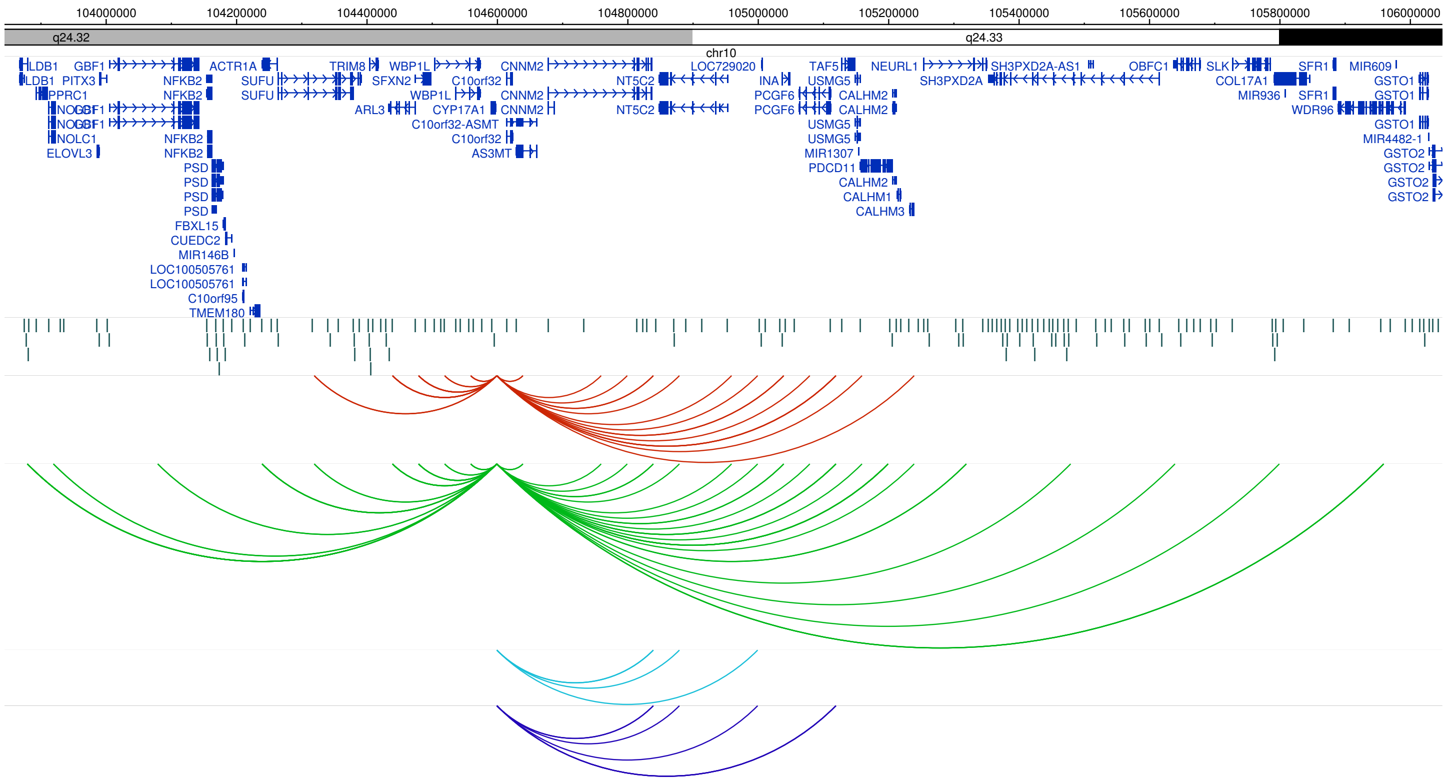

Supplementary Fig. 7: Chromosomal interactions at the AS3MT locus where a cQTL co-localize with SCZ GWAS variants and eQTLs of both AS3MT and WBP1L).

The AS3MT locus is shown with chromosome interaction loops obtained from eHi-C data from fetal and adult brain cortex (red and green respectively) and from Hi-C data from fetal cortical plate and fetal germinal zone (light blue and purple respectively).

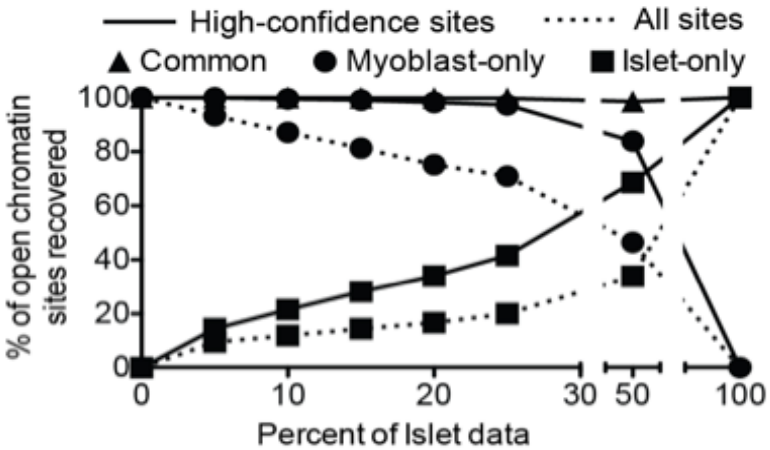

	FACS	Whole tissue
Power to detect chromatin QTLs	Good	Better
Can detect common cell type specific signals?	Yes	Yes
Can we detect rare cell type specific signals?	No	No
Can identify cell type where the DNase signal comes from?	Yes**	No
Mimic natural state of tissue at time of freezing?	Unknown	Yes

**only to the extent of cell sorting

Supplementary Fig. 8: Identification of open chromatin in heterogeneous tissue

(A) DNase-seq data from myoblast and pancreatic islet were mixed in silico. The percentage of tissue-specific and shared chromatin regions are shown. (B) Advantages and disadvantages of FACS sorted nuclei vs. whole tissue analyses.

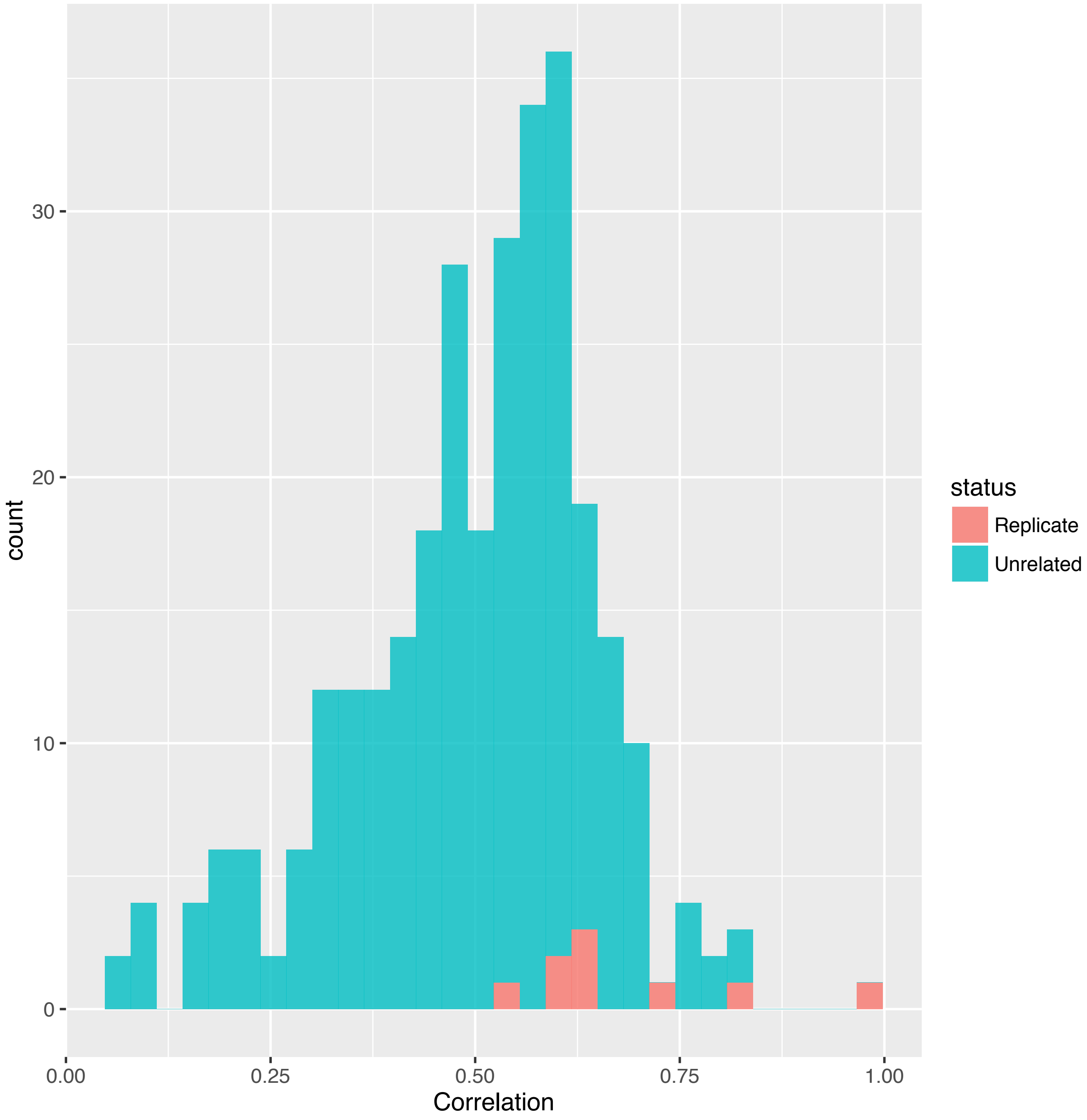

Supplementary Fig. 9: Spearman rank correlations of biological replicates

For biological replicates (9 pairs, red) and all possible combination of the sample with replicates with other samples excluding the replicate samples (blue). Pvalue=1x10^{-4}.

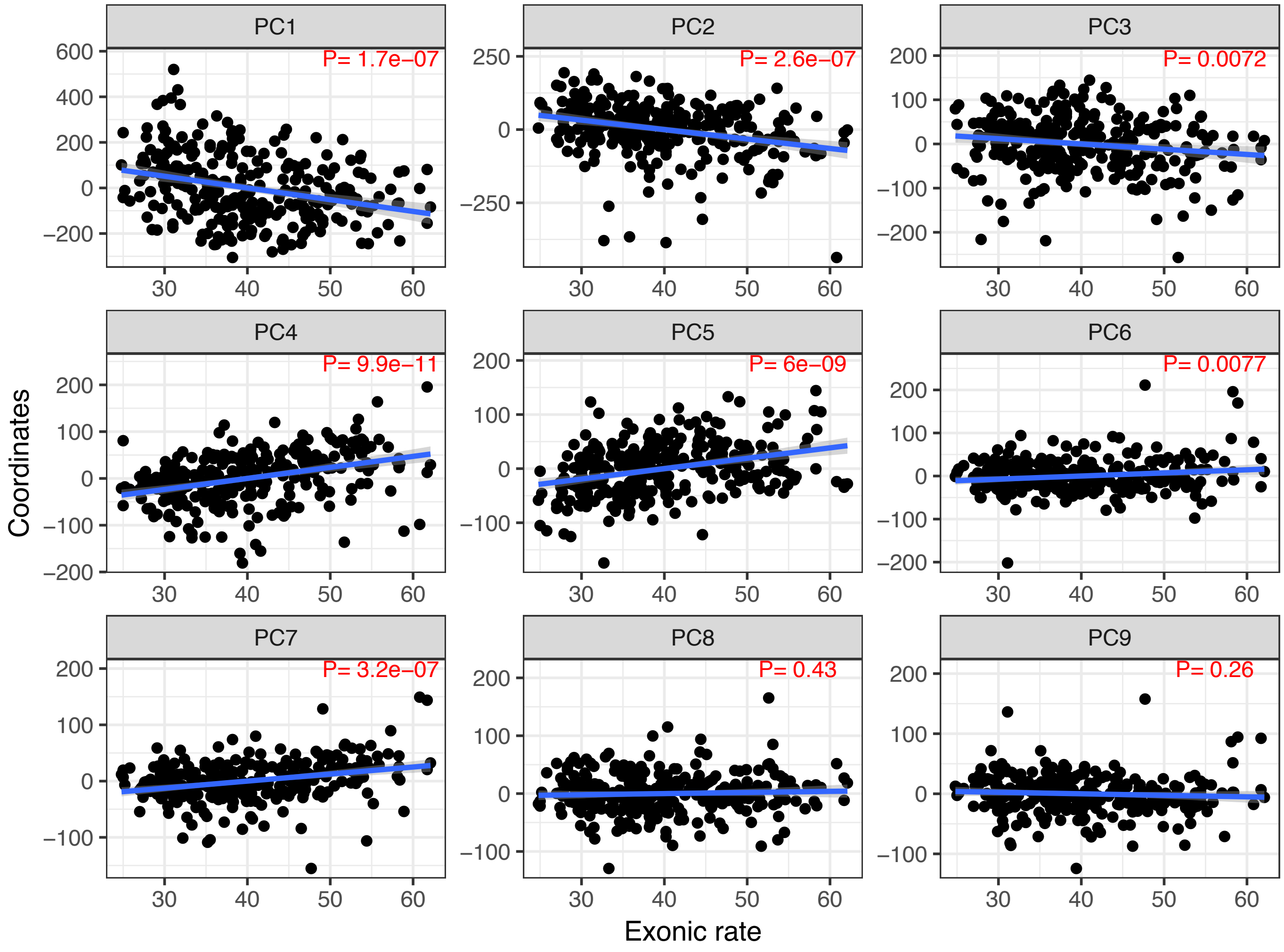

Supplementary Fig. 10: Correlation between ATAC-seq principal components and exonic rate in RNA-seq.

Association between the first 9 principal components of the ATAC-seq quantification matrix and the percentage of reads mapping to exons in RNA-seq (same samples). The association is likely due to an unmeasured confounder that affect both RNA and DNA (sample storage duration for example) and illustrates that multiple genomic assays on the same samples can be used to correct for unwanted variability in one of the genomic assays.

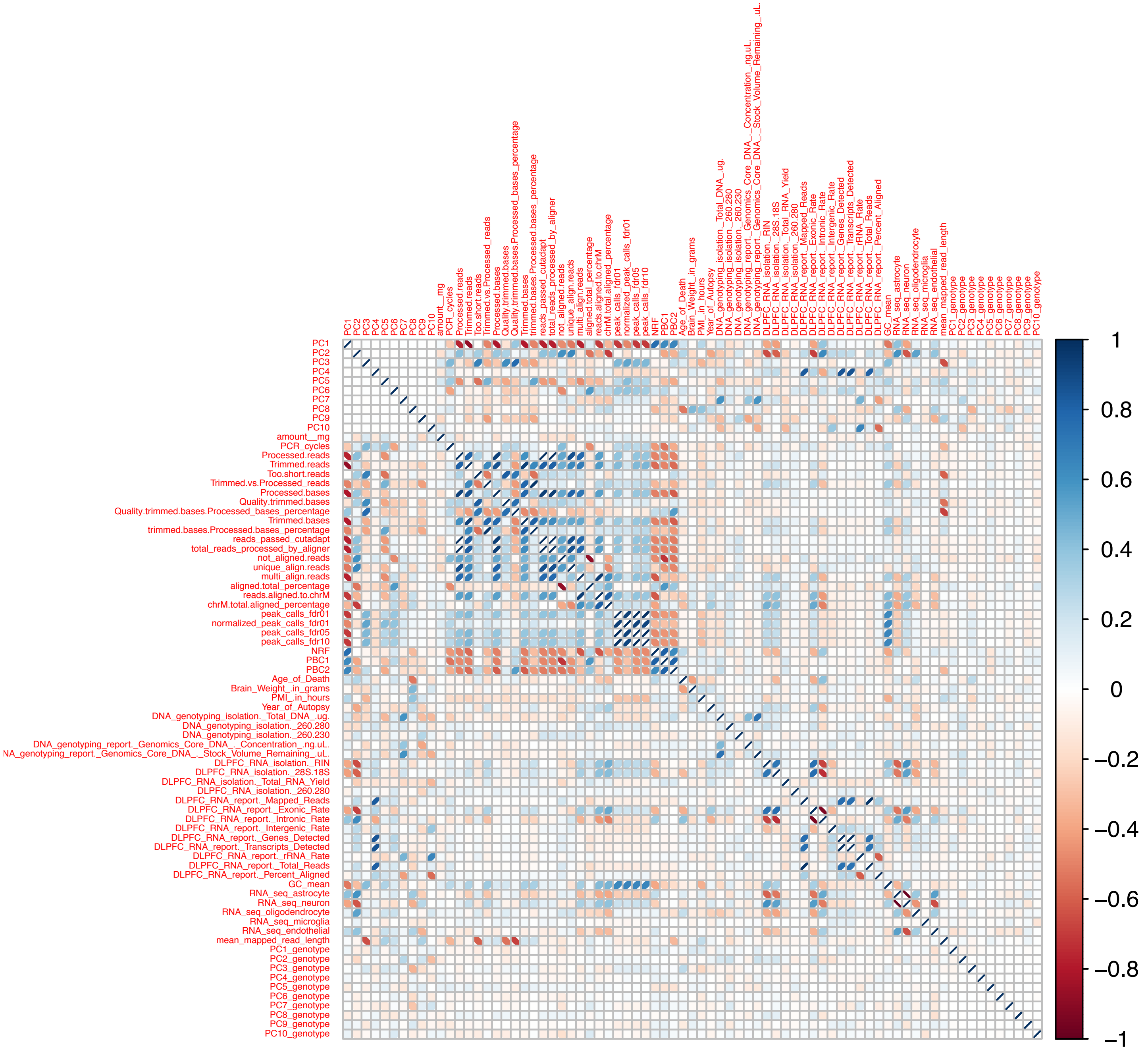

Supplementary Fig. 11: Correlation matrix for imputed numerical metadata in 288 samples.

Pearson correlations were computed between all pairs of imputed numerical meta data variable (65 variables) with the addition of the 10 first principal components of the meta data variables. Blue shows positive correlations and red negative correlations.

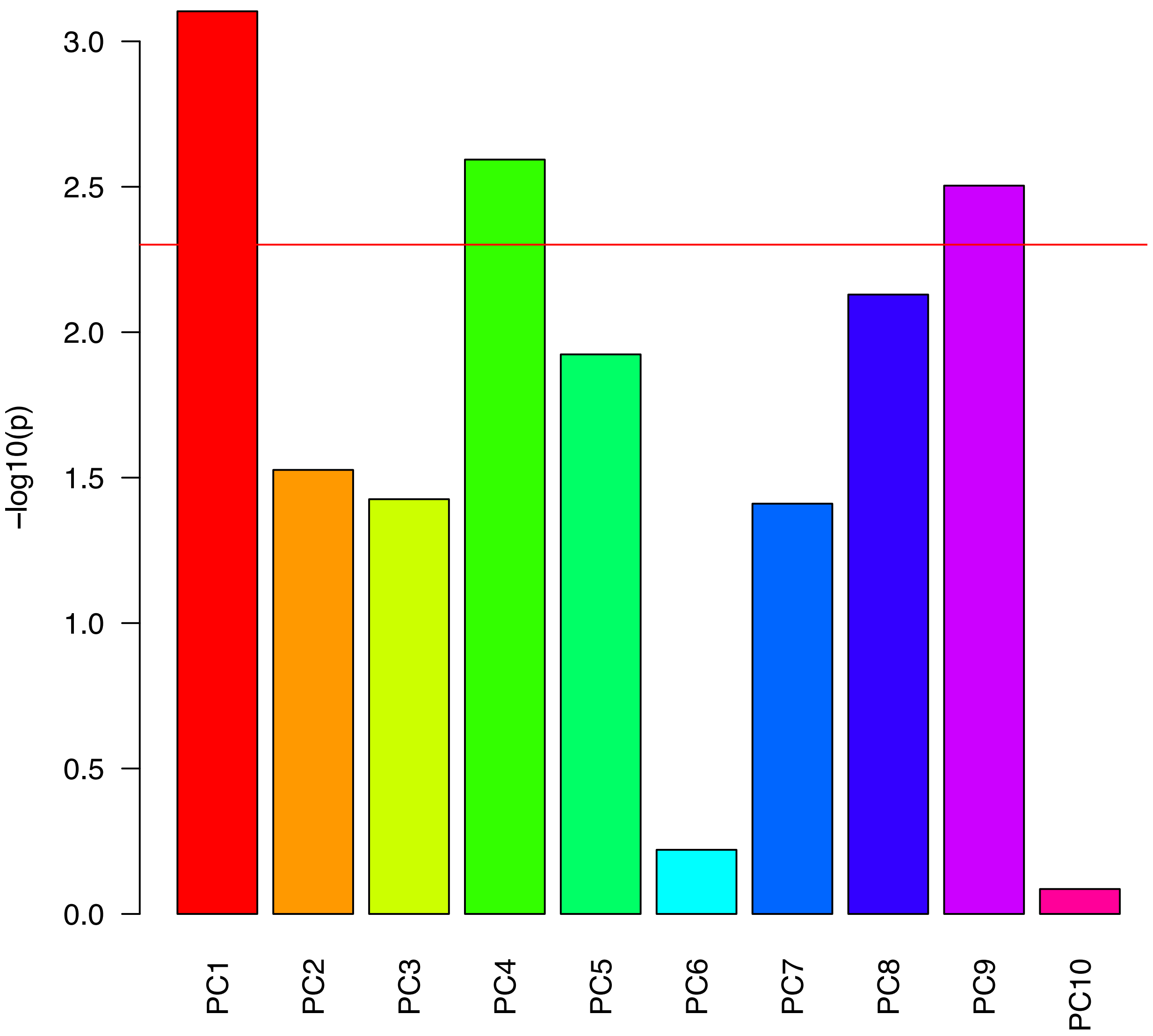

Supplementary Fig. 12: Association of PCs from imputed numerical metadata with diagnosis

The association pvalues were obtained by performing linear regressions. The red bar represents the Bonferonni significance threshold (adjusted pvalue = 0.05)

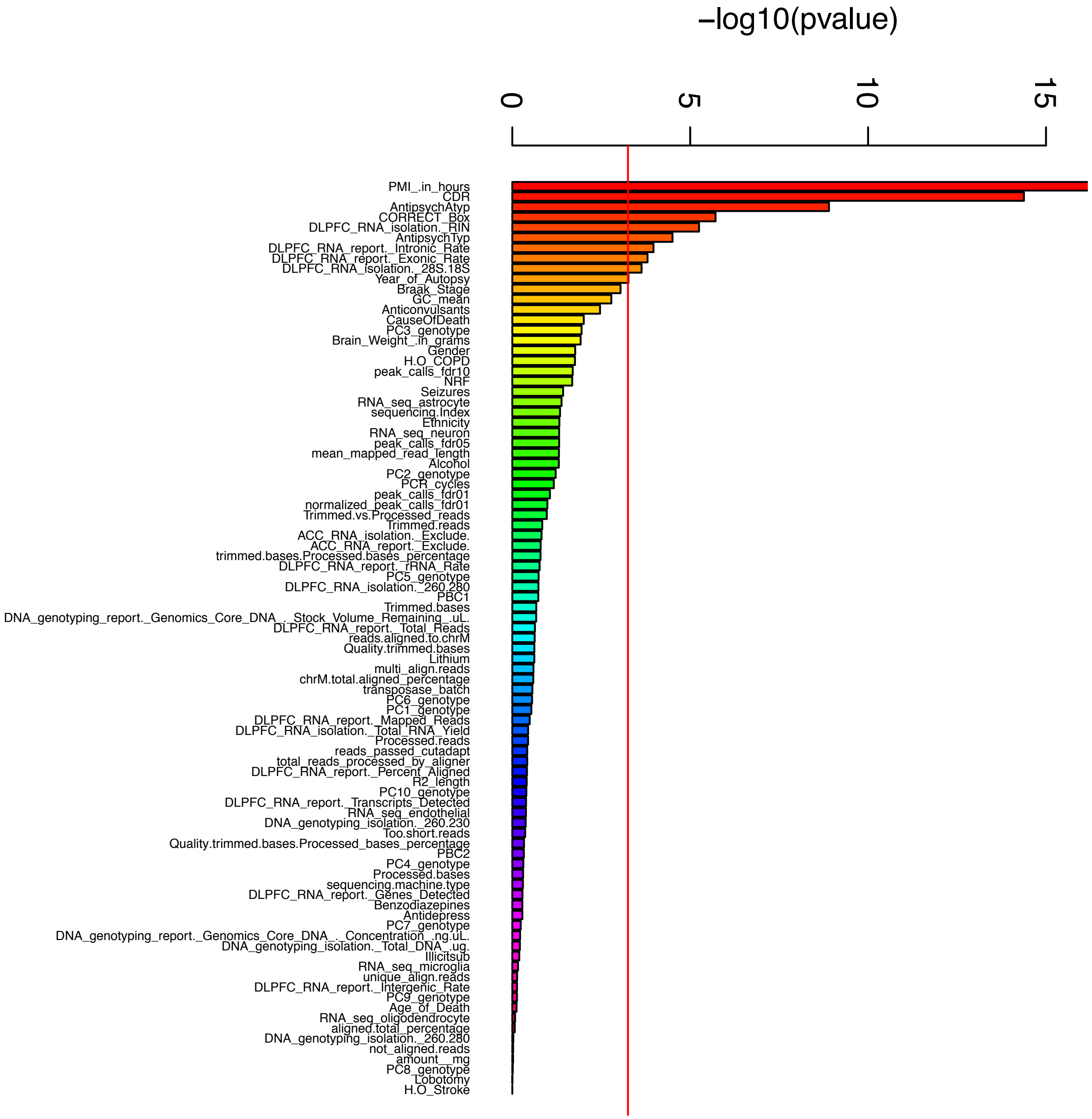

Supplementary Fig. 13: Association of metadata with case-control status

The associations between case-control status (N=135 cases, N=137 controls) and numerical variables were tested using linear regression, while χ^2 tests were performed for categorical variables. The red bar represents the Bonferroni significance threshold (adjusted pvalue = 0.05).

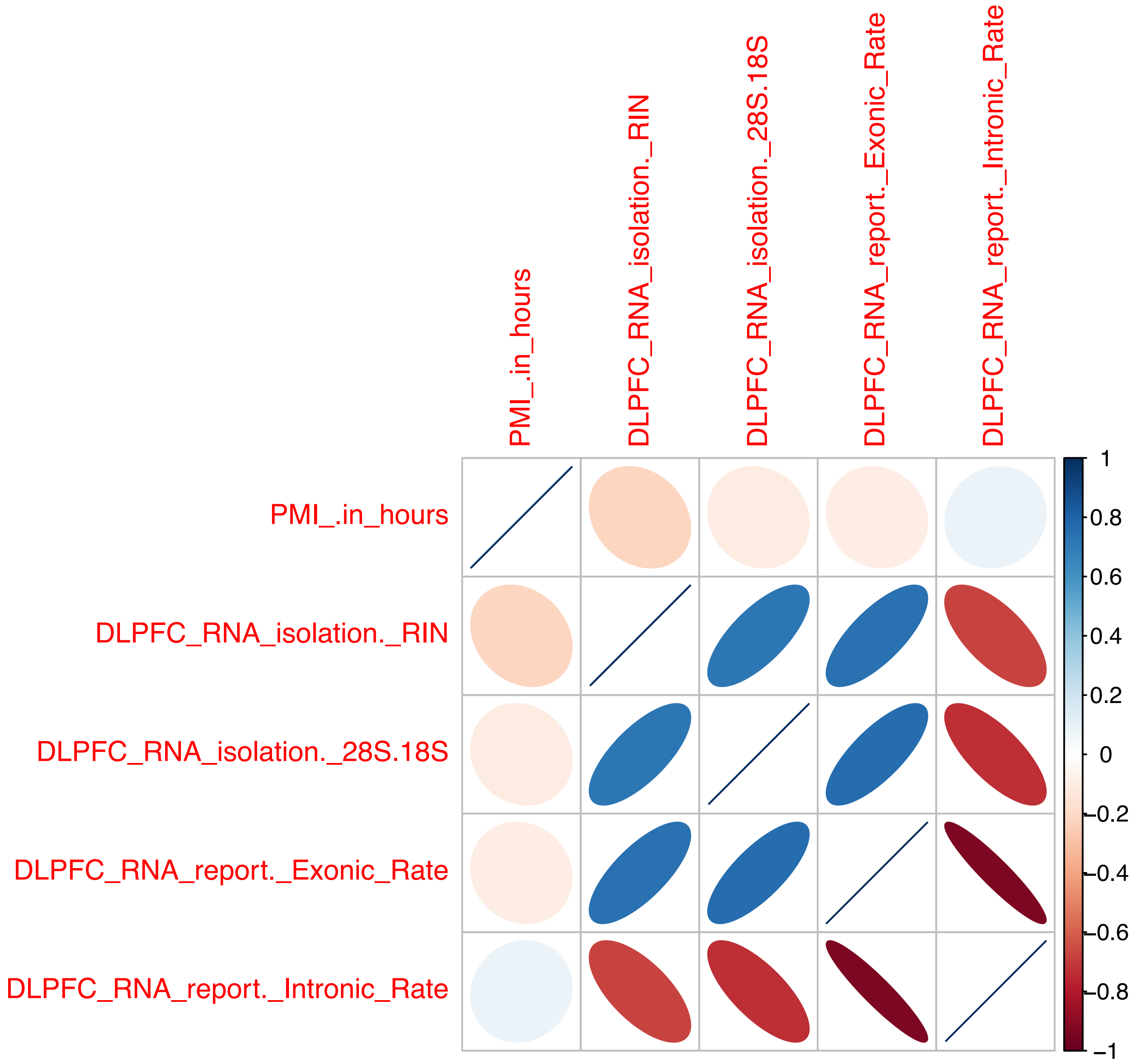

Supplementary Fig. 14: Intercorrelations of numerical metadata significantly associated with case-control status

Pearson correlation between all pairs of numerical meta data variables significantly associated with case/control status are represented (positive correlations in blue, negative correlations in red). Note: The RNA-seq meta data variables are strongly correlated together but are largely independent of the post-mortem interval.

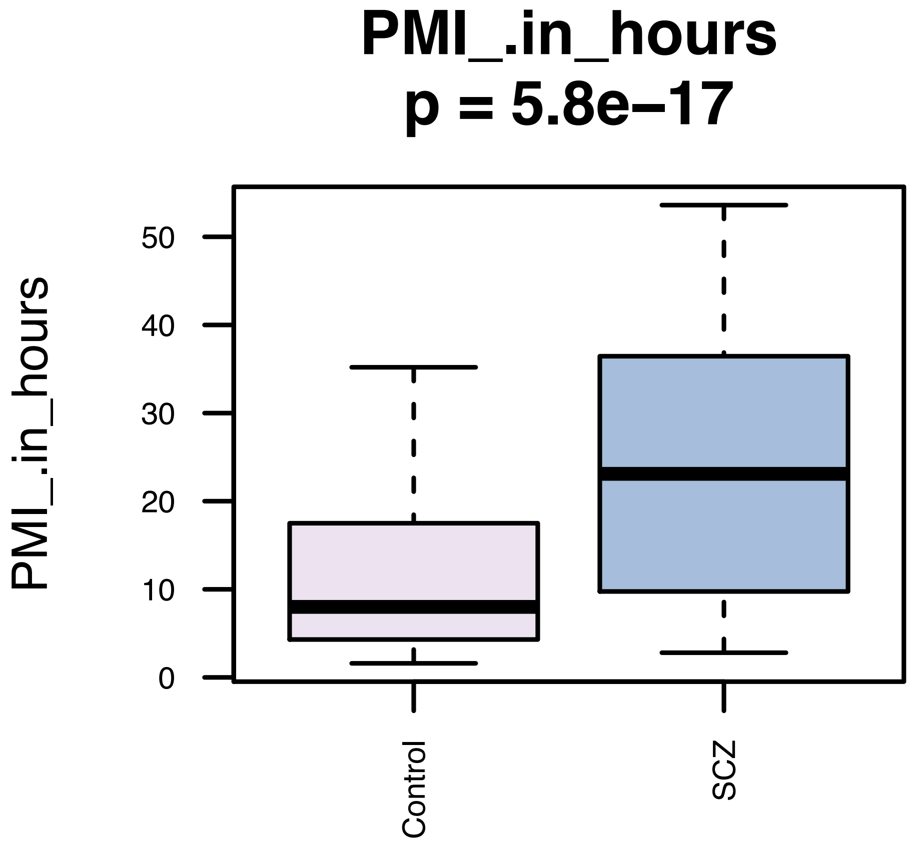

Supplementary Fig. 15: Postmortem interval and case-control status

The P-value was obtained by performing a linear regression between case-control status (N=135 cases, N=137 controls) and post-mortem interval. The boxplots represents the following statistics: median (bolded line), the 1st and 3rd quartiles (bounds of box) and 1.5x the inter-quartile range (whiskers).

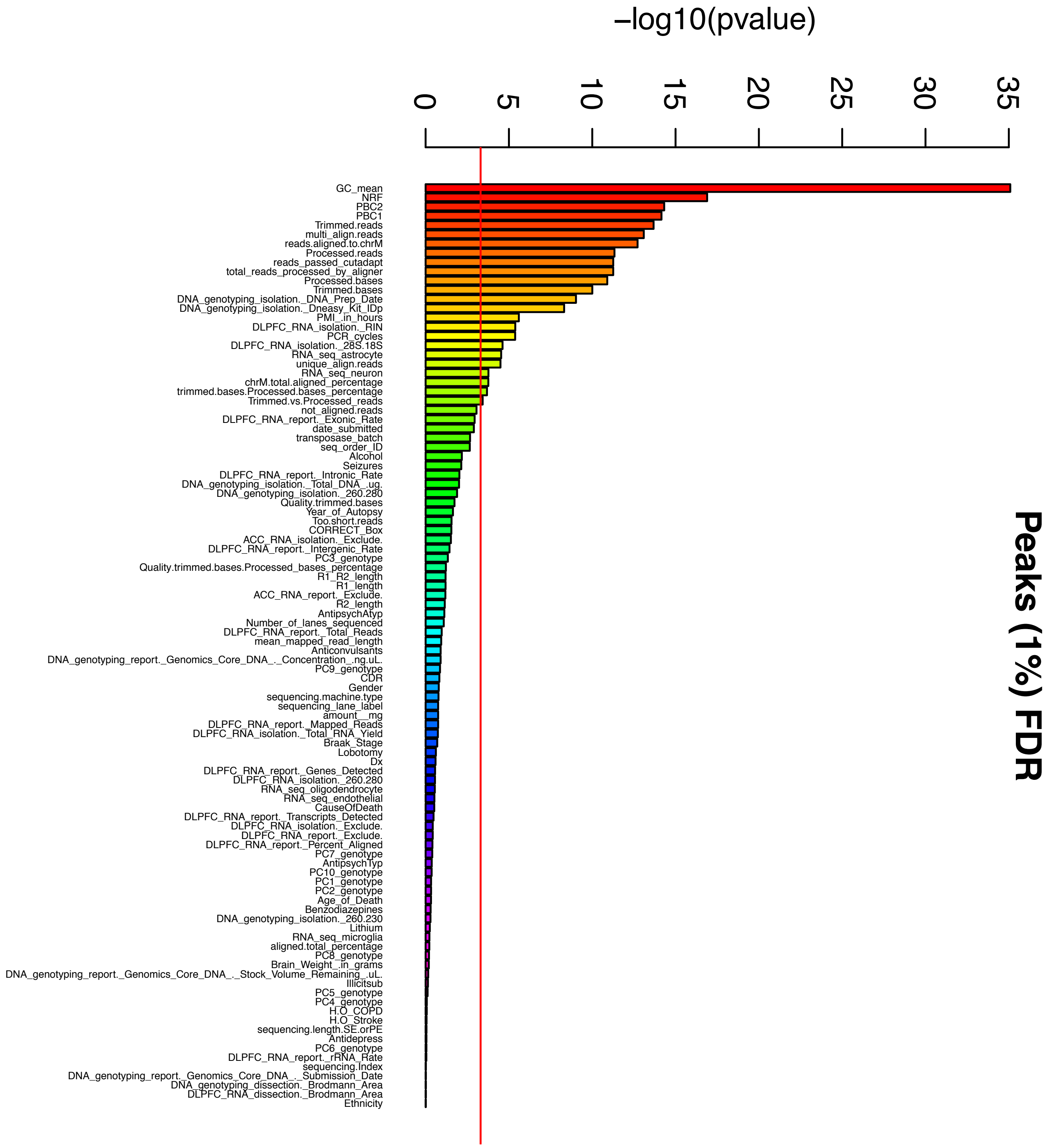

Supplementary Fig. 16: Association of number of ATAC-seq peaks (1% FDR) with metadata

The associations between the number of peaks (1% FDR) and numerical variables were tested using linear regression, while χ^2 tests were performed for categorical variables. The red bar represents the Bonferroni significance threshold (adjusted pvalue = 0.05).

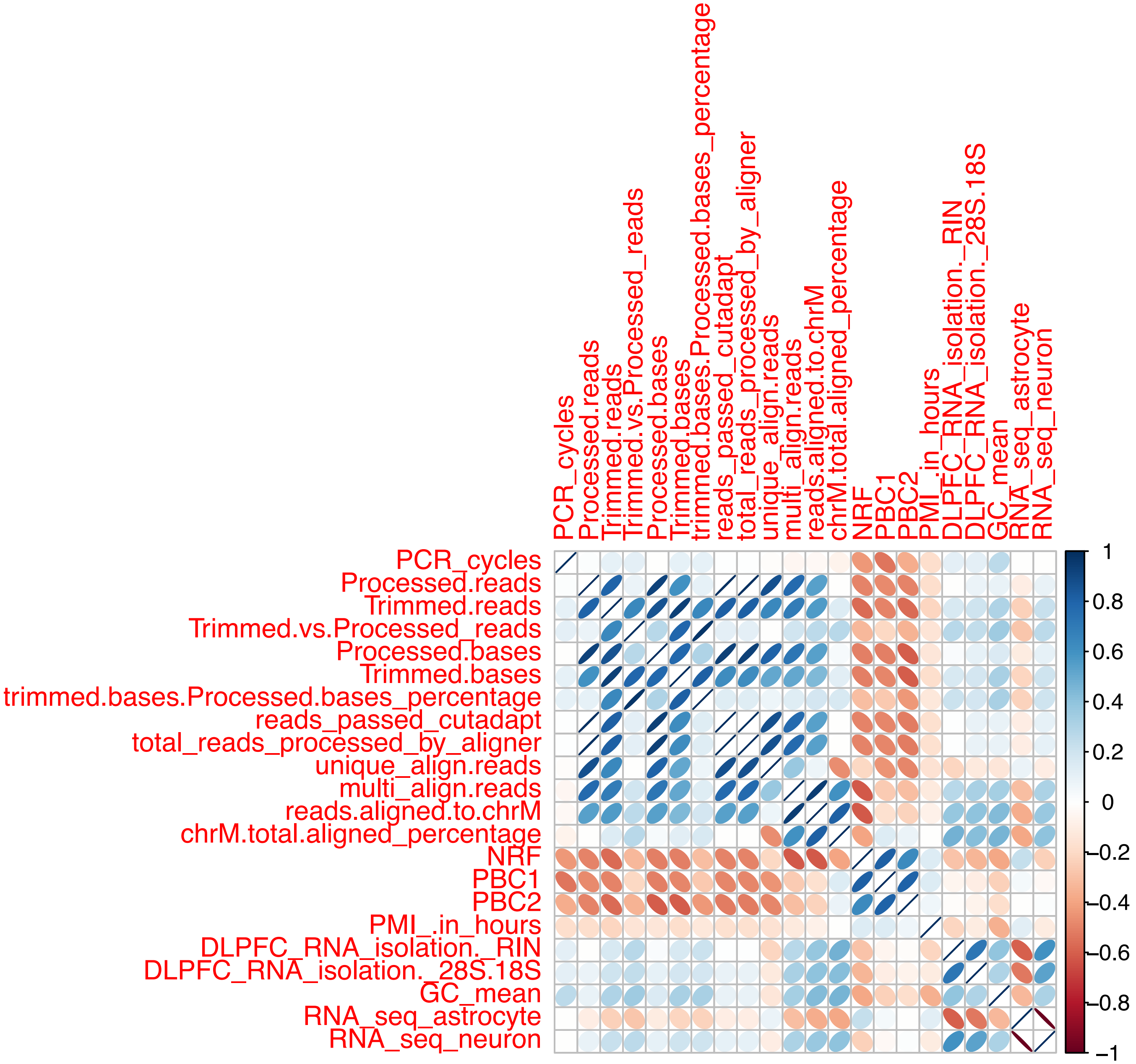

Supplementary Fig. 17: Correlation of technical covariates with number of peak calls

Pearson correlations of 22 numerical meta data variables significantly associated with the number of peak calls (1% FDR). Note: most of the meta data variables significantly associated with the number of peak calls are highly correlated.

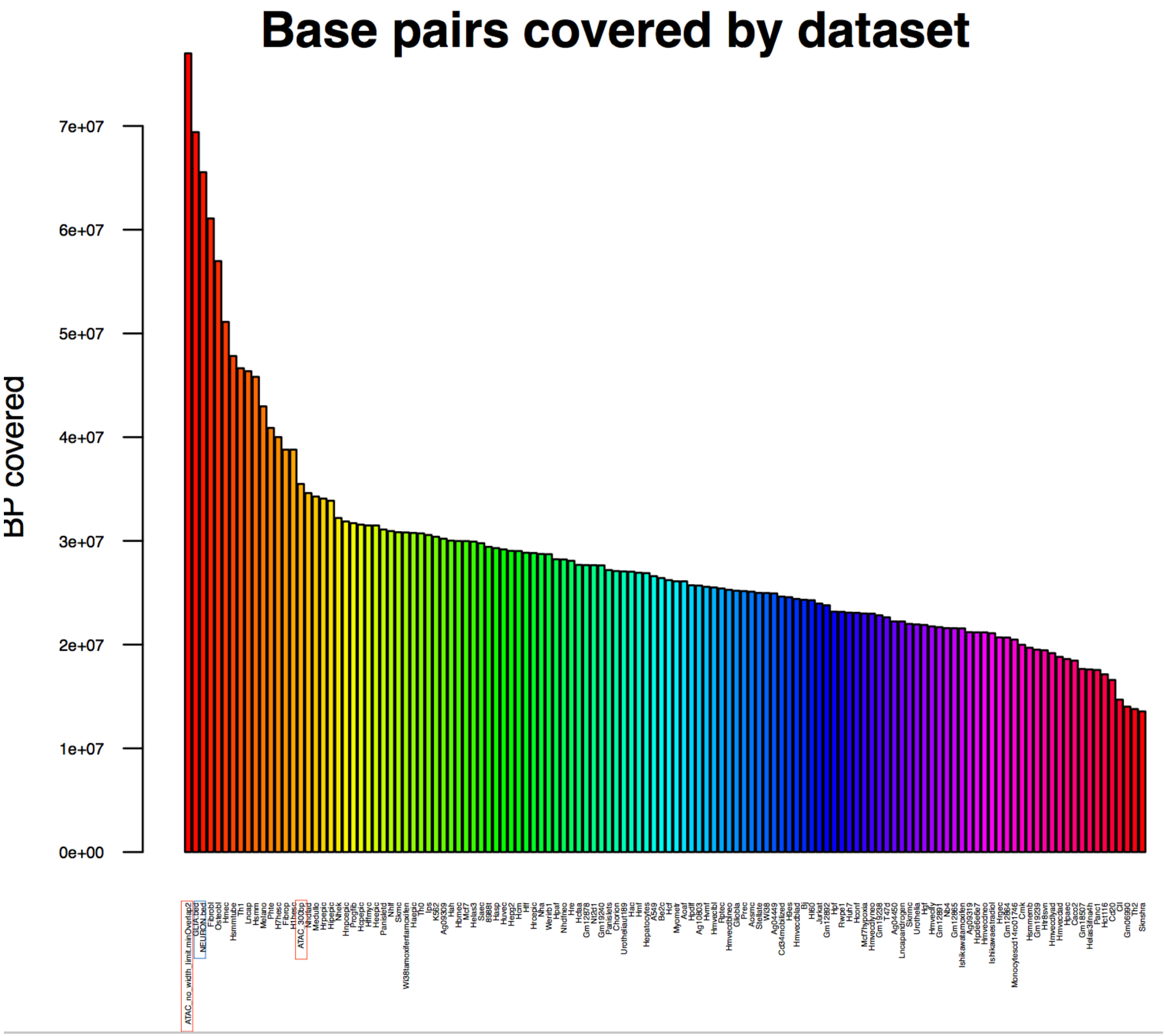

Supplementary Fig. 18: Coverage of ATAC-seq and DNase-seq datasets representing 125 cell types and tissues

The number of base pairs covered by significant peaks in each dataset is shown here. Note: without restricting our ATAC-seq peaks to 300bp, they would be covering significantly more base pairs then ENCODE DNase-seq datasets.

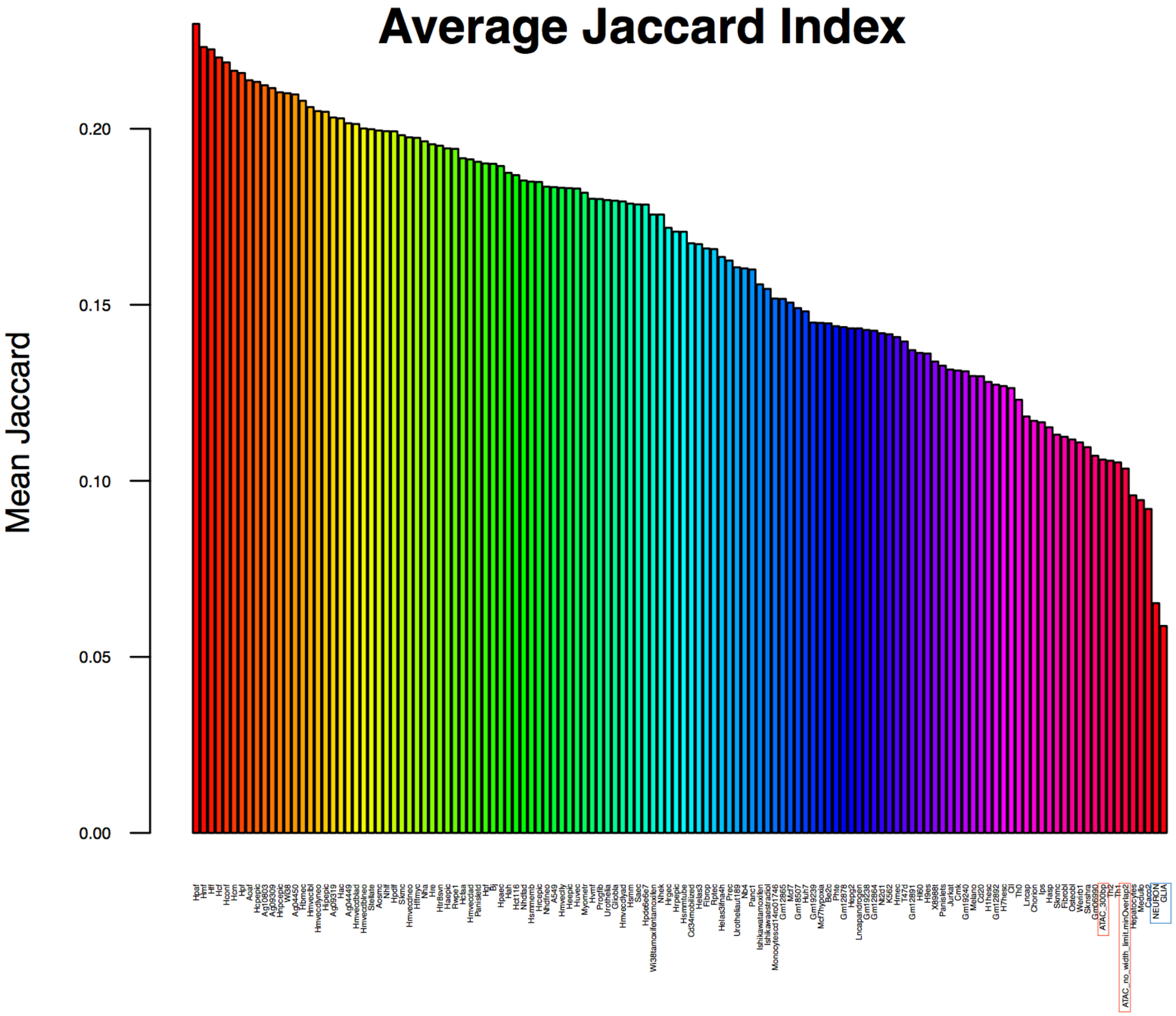

Supplementary Fig. 19: Mean Jaccard index for ATAC-seq and DNase-seq data in 125 cell types and tissues

The Jaccard index (intersection of two data sets divided by their union) was computed between all pairs of ATAC-seq or DNase-seq datasets. The average Jaccard index for each dataset is shown here.

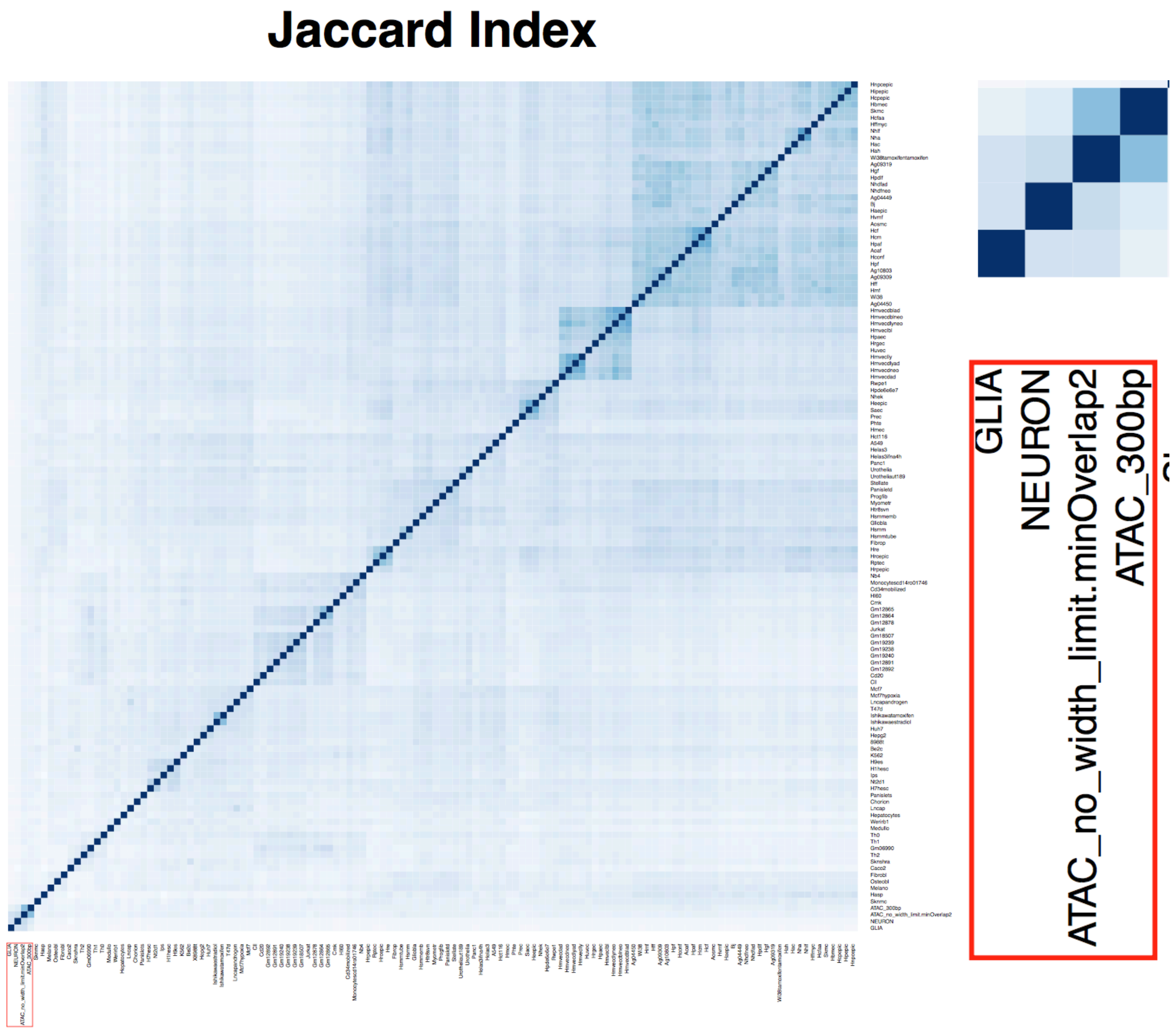

Supplementary Fig. 20: Jaccard index for ATAC-seq and DNase-seq data from 125 cell types and tissues

The Jaccard index (intersection of two data sets divided by their union) was computed between all pairs of ATAC-seq and DNase-seq datasets. White represents a Jaccard index of 0, while dark blue represents a Jaccard index of 1. Panel to right shows correlation similarity to ATAC-seq generated from sorted neuronal nuclei using NeuN+ antibody (NEURON) and NeuN- nuclei (GLIA).

Source	Technique	Tissue	Samples	% Found	Peaks
ENCODE	DHS	Highly diverse	124	5.48	2815428
ENCODE	DHS	CNS-relevant tissue & cell lines	20	13.42	642219
MSSM Roussos	ATAC-seq	Brain, PFC, adult, sorted NeuN-	8	27.97	115216
MSSM Roussos	ATAC-seq	Brain, PFC, adult, sorted NeuN+	8	35.97	115216
Roadmap Project	ATAC-seq	Brain, frontal, fetal	2	14.36	589344
Sullivan-Crawford pilot	ATAC-seq	Brain, frontal, fetal	9	6.27	1118135
Sullivan-Crawford pilot	ATAC-seq	Brain, DLPFC, adult, SCZ cases	9	43.71	99439
University of Chicago	ATAC-seq	Brain, DLPFC, adult, SCZ cases, controls	265	64.71	157660
Sullivan-Crawford pilot	ATAC-seq	Brain, DLPFC, adult, controls	9	72.33	49969

Supplementary Table 1: *Overlap of ATAC-seq data with external samples.*

Overlap of ATAC-seq data from this study with external samples. CNS=central nervous system. DHS=DNase I hypersensitivity. NeuN is a marker for post-mitotic neurons. This table compares our ATAC-seq dataset with regions of open chromatic identified in other experiments including ENCODE, [31] Roadmap Project, [32] a pilot study from Roussos and colleagues, [46] and a separate pilot study from our team (unpublished). Given the widely varying sample sizes and different methods used, we queried the proportion of the open chromatin overlapping with our study. Overlap of open chromatin identified in these other studies was greatest for the most similar studies (DLPFC in adults), somewhat lower in adult samples of sorted neurons, and considerably lower in fetal cortex. Overlap of ENCODE data was low for the full set of highly diverse tissues and cell lines but somewhat higher for the set of CNS-relevant tissues and cell lines. This pattern of results suggests the congruence of our data with those from these other studies.

Gene	PWM	Homer_pvalue	MEME_chip_evalue	Description	
CTCF	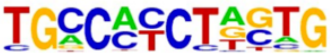	1,00E-114	8,00E-231	Associated with mental retardation/developmental delay; Expressed in DLPFC (CommonMind; Sullivan data); Binds to CHD8 ; Regulates neural development (Hirayama, et al. Cell Rep 2012)	
SP1	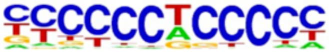	1,00E-57	2,00E-84	mir137 target; REST binding; CHD8 binding	
RFX1	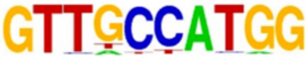	1,00E-37	9,00E-19	REST binding; increase the expression of neuronal glutamate transporter type 3 (Ma et al., JBC 2006)	

Supplementary Table 2: *Enrichment of transcription factor binding motifs in DLPFC ATAC-seq peaks that overlap evolutionarily conserved sequences.*
Shown are top enriched motifs using two different motif enrichment methods, Homer and MEME-chip (background: all ATAC-seq peaks).

1. Fullard, J.F. et al. Open chromatin profiling of human postmortem brain infers functional roles for non-coding schizophrenia loci. *Hum Mol Genet* **26**, 1942-1951 (2017).
2. Robinson, M.D. & Oshlack, A. A scaling normalization method for differential expression analysis of RNA-seq data. *Genome Biol* **11**, R25 (2010).
