# Supplementary material for: Evaluation of chromatin accessibility in prefrontal cortex of individuals with schizophrenia
Source: Nat Commun. 2018 Aug 7;9:3121. doi: 10.1038/s41467-018-05379-y (PMC6081462; doi:10.1038/s41467-018-05379-y)
Supplement: Supplementary file 3 — Description of Additional Supplementary Files [file 41467_2018_5379_MOESM3_ESM.pdf]

Description of Additional Supplementary Files

Supplementary Data 1: Differential chromatin analysis results for age at death.

Supplementary Data 2: GREAT analysis of differentially accessible peaks for age at death (9,396 peaks, 20% FDR)

Supplementary Data 3: Differential chromatin analysis results for post-mortem interval

Supplementary Data 4: GREAT analysis of differentially accessible peaks for post-mortem interval (2,328 peaks, 20% FDR)

Supplementary Data 5: Differential chromatin analysis results for diagnosis (control vs schizophrenia).

Supplementary Data 6: GREAT analysis of differentially accessible peaks for diagnosis (control vs schizophrenia, top 1000 peaks).

Supplementary Data 7: Meta-data variables associated with samples used in this study.
